# The Assessment of the Prevalence of Impacted Third Molars, Its Relation to Space Available for Eruption, and Its Effect on the Adjacent Second Molar in Adults From 18 to 65 Years of Age Using Orthopantomograms: A Retrospective Cross-Sectional Study

**DOI:** 10.7759/cureus.78608

**Published:** 2025-02-06

**Authors:** Janhavi D Modi, Shikha A Gala, Himanshi A Dave, Shreyas H Gupte, Shruti Singh

**Affiliations:** 1 Oral and Maxillofacial Surgery, Dr. G.D. Pol Foundation's Y.M.T. Dental College and Hospital, Navi Mumbai, IND; 2 Dentistry, Dr. G.D. Pol Foundation's Y.M.T. Dental College and Hospital, Navi Mumbai, IND

**Keywords:** angulation of impaction, eruption space ratio, impacted third molar, level of impaction, nerve involvement, orthopantomogram, radiographic study, second molar caries, sinus approximation, third molars

## Abstract

Introduction

Third molar impaction surgeries are part of the most common minor oral surgeries performed by maxillofacial surgeons. In spite of being part of the everyday evaluation, the importance of preoperative planning and intraoperative and postoperative complications cannot be overlooked. This study aims to evaluate the prevalence, patterns, available eruption space, and impact of third molar impaction on the adjacent second molar in an adult population.

Methods

A retrospective observational cross-sectional study was conducted over a total of 380 panoramic radiographs from patients aged 18-65 years, which was analyzed to assess the incidence, angulation, and space available for the eruption of impacted third molars and their effects on the adjacent second molars. Orthopantomograms (OPGs) from January 2023 to December 2023 were included in the study. A total of 1280 third molars from 380 OPGs were studied encompassing an extensive range of demographics.

Results

The results showed that 54.7% (208/380) of the studied population had at least one impacted third molar, with a higher prevalence in the mandibular arch. Vertical impaction was the most common pattern (224/419, 53.46%), followed by mesioangular (92/419, 21.95%), horizontal (85/419, 20.28%), and distoangular (18/419, 4.29%) impactions. Notably, the study found a relevant association between impacted third molars and the development of distal caries in the adjacent second molar (76/419, 18.13%). These findings highlight the importance of early radiographic evaluation of impacted third molars to prevent potential damage to adjacent teeth and inform clinical decisions regarding the management of such cases. The study also highlights that there is a significant amount of third molar impaction seen in women (211/380, 55.52%) compared to men, and the age group range with the highest incidence of third molar impaction is 18-25 years (399/1280, 31.20%).

Additionally, a significant number of mandibular impacted third molars present with some level of nerve involvement, whereas more than 80% of maxillary impacted third molars showed sinus approximation. This study also presents a novel concept of "eruption space ratio," which is the ratioof the mesiodistal width of the crown of the third molar to the space available posterior to the second molar for eruption. When it was compared to the angulation, a substantial link was seen, although none was seen in relation to the level of impaction.

Conclusion

This study underlines the need for proactive dental care in populations with a high prevalence of third molar impaction, aiming to mitigate the risks to the adjacent second molars and to eliminate possible postoperative complications through timely intervention.

## Introduction

Third molars being the last to erupt into the oral cavity are the most frequently found impacted teeth in human beings [1]. Multiple factors including human development and evolution over the past few decades have resurfaced as the etiological factors of impactions. Smaller jaw size, dense overlying bone, improper eruption of the adjacent tooth, and arch-length discrepancies are a few such factors that can result in a decrease in available space for the eruption of the third molar [2].

Impacted wisdom teeth are commonly seen in dental practice, with a prevalence ranging from 9.5% to 68% across populations, more than 50% of patients having at least one impacted third molar, and the mandibular third molars being the most frequently affected [2]. Mesioangular impaction is one of the most common patterns of impaction seen, closely followed by horizontal and vertical types of impaction [2].

These impactions are commonly associated with complications such as food lodgement, pericoronitis, and caries posing a threat to the adjacent healthy teeth [3]. There is also a significant relationship between the carious involvement of the second molar adjacent to the impacted third molars [4]. Thus, to understand the current prevalence of impacted molars and the need for extractions and to assess the space available for extraction, with no data available in the literature assessing the relation between the angulation and level of impaction, a hypothesis was formulated.

Our retrospective study aimed to compare and assess the prevalence and pattern of impacted third molars and their effect on the adjacent second molar. This study also aimed to understand the relation, if any, between the angulation and level of impaction with respect to the space available for eruption.

## Materials and methods

Retrospective radiographic evaluation from orthopantomograms (OPGs) of 380 adult patients (18-65 years of age) was studied using the Strengthening the Reporting of Observational Studies in Epidemiology (STROBE) guidelines for the retrospective observational cross-sectional study between January 2023 and December 2023 at Y.M.T. Dental College and Hospital, Navi Mumbai. A total of 1280 third molars from these OPGs were studied. The study was reviewed and approved by the Institutional Review Board (IRB) of Dr. G.D. Pol Foundation's Y.M.T. Dental College and Hospital (IRB number: 194/IRB/YMTDC24).

Inclusion criteria and exclusion criteria

**Figure 1:**
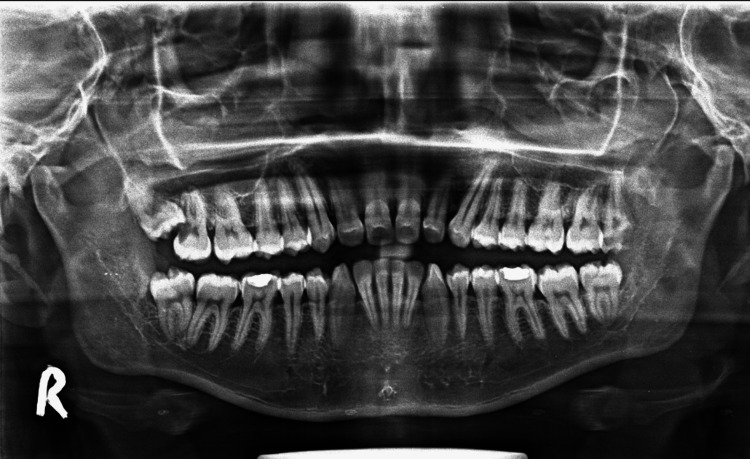
An orthopantomogram that was included in the study, which also shows sinus approximation in impacted maxillary third molar

OPGs with the presence of a third molar with any angulation and level of impaction with an adjacent second molar and their related data were tabbed. Excluded from the study were OPGs with incomplete root formation of the third molars, any pathology involving second and third molars (not including any caries-related pathologies), any OPG suggestive of genetic/syndromic disorders, and the presence of incomplete records or poor quality of OPGs (Figure 1).

Data collection and analysis

The formula used for determining the sample size is the single proportion formula: n=d2Z2⋅p⋅(1−p), where n is the required sample size, Z is the z-score corresponding to the confidence level (for 95% confidence, Z=1.96), p is the estimated proportion (here, p=0.45) and d is the desired margin of error (here, d=0.05). Substituting the values, n=(0.05)2(1.96)2×(0.45)×(1−0.45), n=0.00253.8416×0.45×0.55, and n=0.00250.9503=380.12. This rounds to 380.31, which aligns with our calculation.

Third molars that do not reach the functional occlusal level were classified as impacted and analyzed for the angulation, level of impaction, and the type of nerve involvement in mandibular and sinus approximation (if the roots were ≤2 mm from the maxillary sinus lining) in maxillary molars (Figure 1), with the presence of caries in adjacent second molars also being noted.

**Figure 2:**
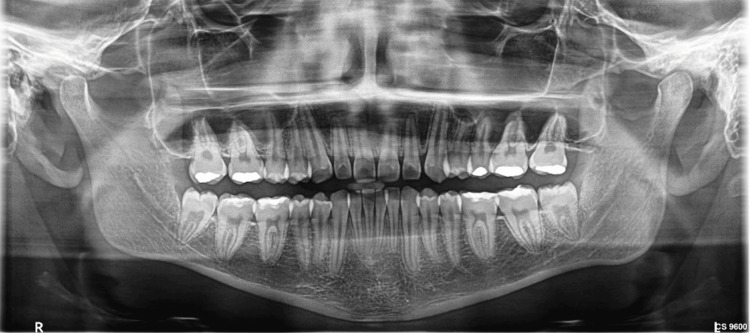
An orthopantomogram showing nerve involvement

The angulation was evaluated using Quek's adaptation of Winter's classification. The Pell and Gregory classification was implemented to ascertain the degree of impaction, whereas the Rood and Shehab classification was utilized to assess the type of nerve involvement (Figure 2).

Eruption space ratio

**Figure 3:**
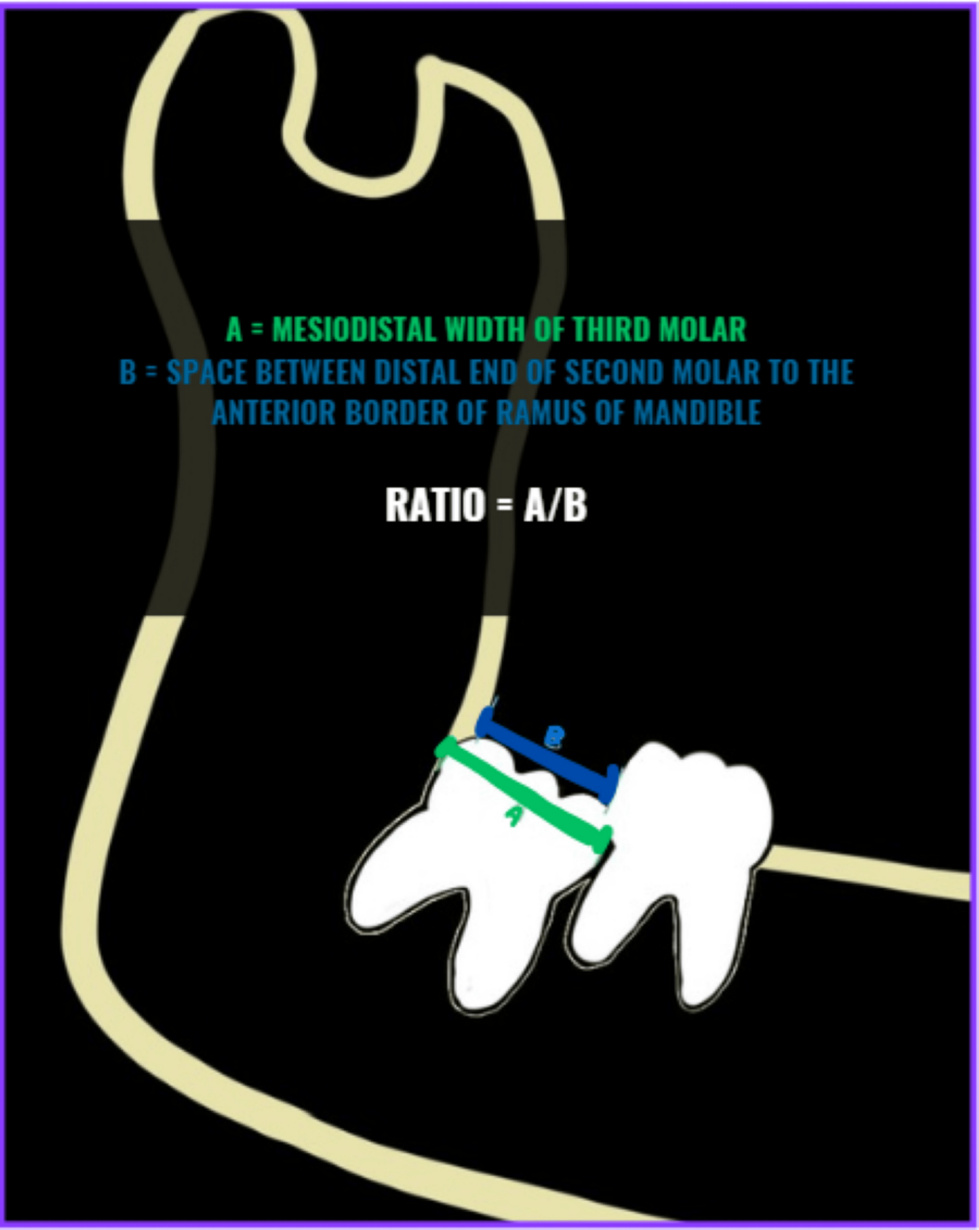
Schematic image showing the measurements of the eruption ratio Image credit: Himanshi Dave

The eruption space ratio (Figure 3) was calculated by measuring the mesiodistal width of the impacted third molar and dividing it by the distance from the distal end of the adjacent second molar to the anterior border of the ramus. We then correlated this data to the angulation and impaction level noted in the third molars.

The collected comprehensive data was compiled on an MS Office Excel worksheet (Microsoft Corp., Redmond, WA) and was subjected to statistical analysis using an appropriate package such as SPSS software (IBM Corp., Armonk, NY) and studies to understand third molar impactions. Descriptive statistics, such as the frequency (n) and percentage (%) of categorical data, as well as the mean and standard deviation of numerical data in each group, are presented. The comparison of response frequencies with independent variables is conducted using the chi-square test. We are maintaining a 5% alpha error, 20% beta error, and 80% power, with a significance level of p<0.05.

## Results

**Figure 4:**
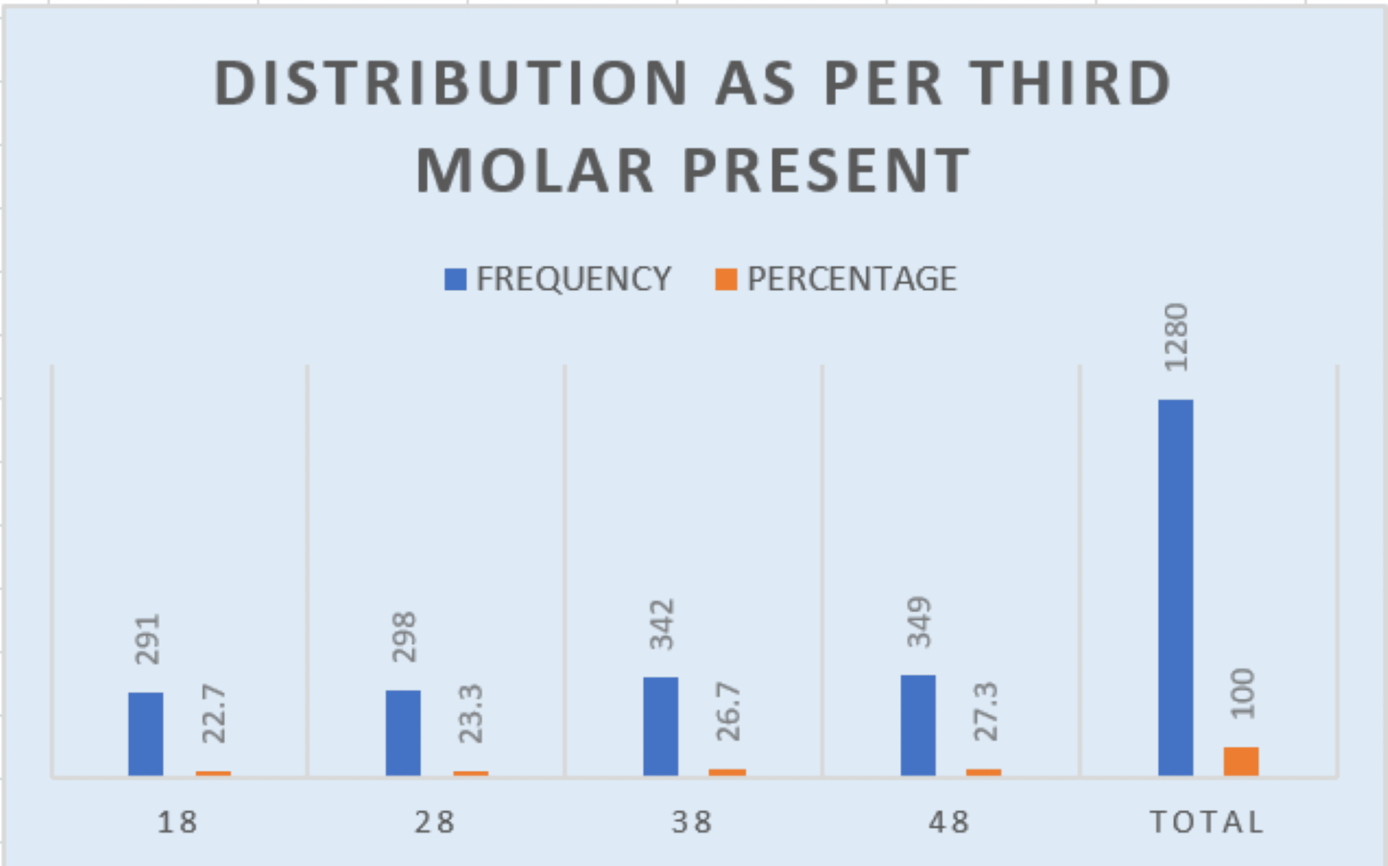
Graph describing the specific third molar distribution in the studied sample size

Three hundred eighty OPGs with 1280 third molars were analyzed. The analysis revealed that 208 of 380 OPGs (54.7%) had at least one impacted third molar. Among 380 OPGs, a total of 1280 third molars were studied (distribution of third molars present): of all the present third molars, 48 had the highest frequency at 349 (27.3%), followed by 38, 28, and 18 (Table 1 and Figure 4).

**Table 1:** The specific third molar distribution in the studied sample size

	Value	Frequency	Percentage
	18	291	22.7
	28	298	23.3
	38	342	26.7
	48	349	27.3
Total		1280	100

Distribution according to age

The complete sample group was allocated to different age cohorts, i.e., 18-25, 26-35, 36-45, 46-55, and 55-65, to study the incidence of third molars. The maximum number of third molars was present in the age group of 18-25: 399 (31.2%) (Table 2). According to the chi-square test, this relation between age and third molars present was found to be highly significant (p=0.00).

**Table 2:** Third molars present in each quadrant in relation to age group Statistical analysis: chi-square=45.404, df=20, and P value=0.001 df: degrees of freedom

Third molars present	18-25 years	26-35 years	36-45 years	46-55 years	56-65 years	Total
18	96	71	59	39	26	291
28	95	77	59	40	27	298
38	103	81	68	52	38	342
48	105	84	69	53	38	349
Total	399	313	255	184	129	1280

Two hundred thirty-two (61%) patients had all four third molars of which 122 (52.58%) women and 110 (47.47%) men showed the same (Table 3). There was one rare case of a male having five third molars recorded, and the fifth third molar could be considered supernumerary but had the morphology of a molar. A statistically strong correlation was found between gender and the number of third molars present, with women accounting for 211 (54.6%) of third molars observed in this study.

**Table 3:** The number of third molars present in relation to gender Statistical analysis: chi-square=24.807, df=8, and P value=0.002 df: degrees of freedom

Number of third molars present	Female	Male	Total	Percentage
1	13	7	20	5.26
2	30	23	53	13.94
3	46	28	74	19.47
4	122	110	232	61.05
5	0	1	1	0.26
Total	211	169	380	100

Distribution of impaction

Of the third molars studied, 419 (32.8%) were impacted. The highest frequency of impaction (155, 36.99%) was seen in 48, with more impactions observed in the mandible than in the maxilla (Table 4).

**Table 4:** The specific third molars impacted

Values	Count	Percentage
18	59	14.08115
28	58	13.84248
38	147	35.08353
48	155	36.99284
Total	419	100

**Figure 5:**
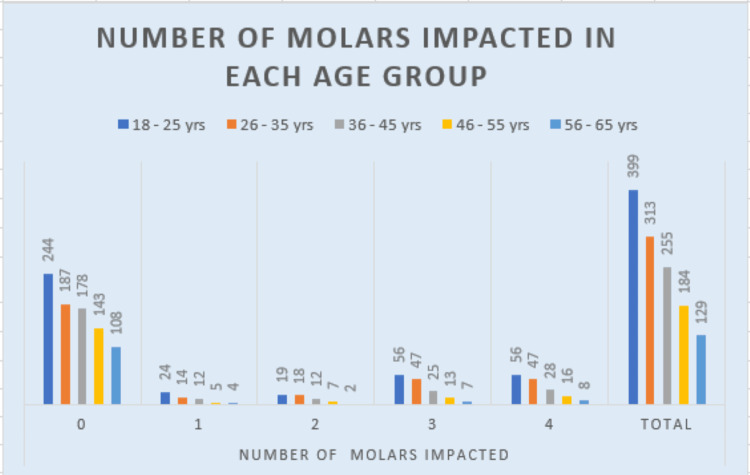
Graph showing the distribution of the number of third molars in each age group

A strong correlation was found between age groups and impacted third molars (p<0.01). The highest frequency of impacted third molars was 154 (36.75%) in the 18-25 age group (Table 5 and Figure 5). When comparing the number of impactions between men and women, no statistical significance was observed.

**Table 5:** The distribution of the number of third molars in each age group Statistical analysis: chi-square=45.404, df=20, and P value=0.001 df: degrees of freedom

Number of third molars impacted	18-25 years	26-35 years	36-45 years	46-55 years	56-65 years	Total
0	244	187	178	143	108	860
1	24	14	12	5	4	59
2	19	18	12	7	2	58
3	56	47	25	13	7	148
4	56	47	28	16	8	155
Total	399	313	255	184	129	1280

Distribution of the angulation of impaction

**Figure 6:**
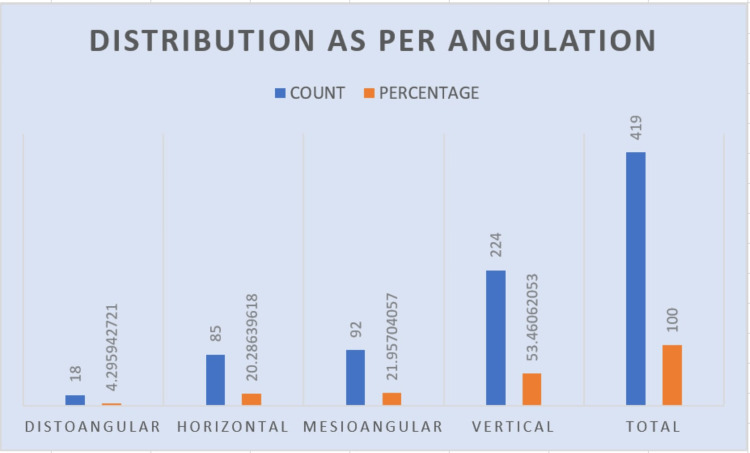
Graph describing the distribution of angulation of impacted molars

The angulation of impactions were assessed as per the adaptation of Winter's classification. Within the dataset, maximum impactions showed vertical impactions (224, 53.46%), followed by horizontal (85, 20.28%) and mesioangular (92, 21.95%) impactions with similar frequency. Eighteen (4.29%) showed distoangular impactions (Figure 6).

With 87 (53.46%) vertical impactions documented in the age group of 18-25, the relation between the angulation of impactions and age group also showed a notable association (Table 6).

**Table 6:** The distribution of angulations among the impacted third molars in relation to age groups Statistical analysis: chi-square=57.976, df=16, and P value=0 df: degrees of freedom

	18-25 years	26-35 years	36-45 years	46-55 years	56-65 years	Total	Percentage
Distoangular	5	6	5	1	1	18	4.29
Horizontal	24	19	22	13	7	85	20.28
Mesioangular	38	29	10	10	5	92	21.95
Vertical	87	72	40	17	8	224	53.46
Total	154	126	77	41	21	419	100

**Figure 7:**
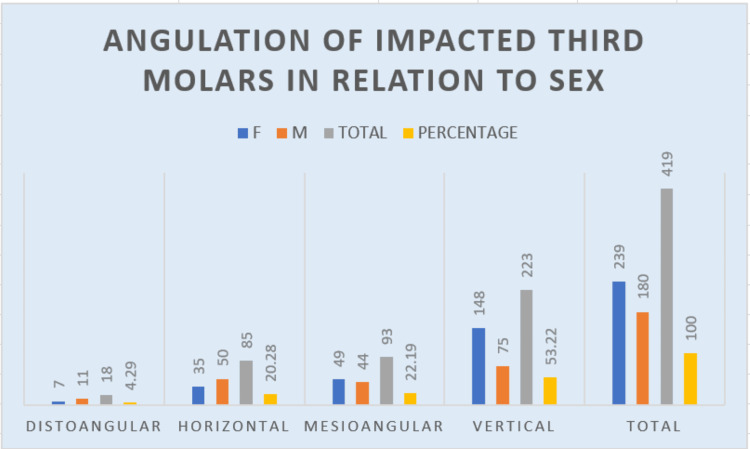
Graph describing the angulation of impacted molars to gender

Within the female impaction cases, a strikingly higher prevalence of 148 (61.9%) vertical impactions was seen, followed by mesioangular, horizontal, and then distoangular. In men, 75 (41%) impactions were vertical, followed by horizontal, mesioangular, and distoangular. A significant connection between the angle of impaction and sex was also noted, with vertical impactions being the most common (Figure 7).

Level of impaction

**Figure 8:**
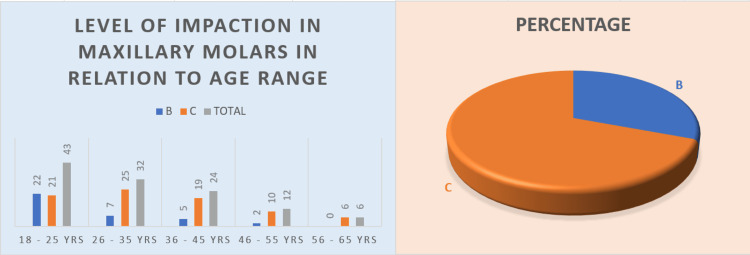
Graph describing the level of impaction in maxillary molars with respect to age

**Figure 9:**
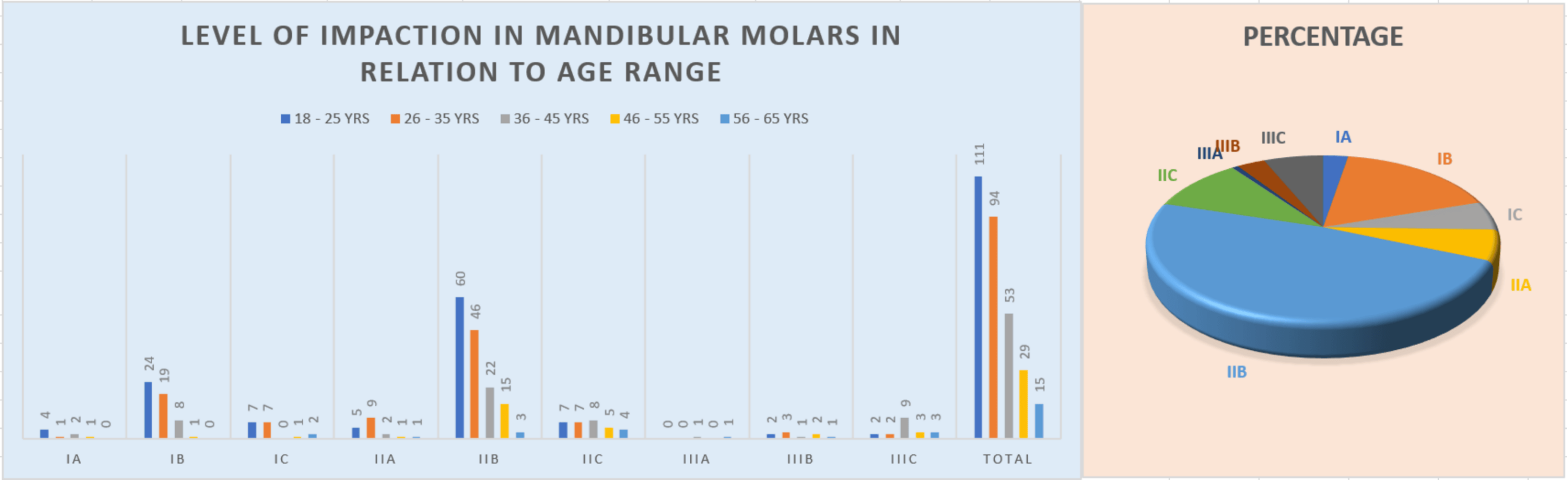
Graph describing the level of impaction in mandibular molars with respect to age

Following the Pell and Gregory classification, 146 (48.18%) mandibular molars were noted to be impacted at level IIB and 81 (69.82%) at level C maxillary impactions (Figure 8), making them the most common level. There was a significant correlation with age with 60 (19.8%) level IIB impactions being observed in individuals from 18 to 25 years, followed by IB level impactions in mandibular molars. Level IIIA was scarcely observed with only two such instances. Individuals in the 18-25-year cohort showed high frequencies in almost all levels except for IIC and IIIC, which were most common in individuals aged 36-45 (Table 7 and Figure 9).

**Table 7:** The level of impaction in mandibular molars with respect to age Statistical analysis: chi-square=108.316, df=44, and P value=0 df: degrees of freedom

Level of impaction (mandible)	18-25 years	26-35 years	36-45 years	46-55 years	56-65 years	Total	Percentage
IA	4	1	2	1	0	8	2.64
IB	24	19	8	1	0	52	17.21
IC	7	7	0	1	2	17	5.62
IIA	5	9	2	1	1	18	5.96
IIB	60	46	22	15	3	146	48.34
IIC	7	7	8	5	4	31	10.26
IIIA	0	0	1	0	1	2	0.66
IIIB	2	3	1	2	1	9	2.98
IIIC	2	2	9	3	3	19	6.29
Total	111	94	53	29	15	302	100

Nerve involvement

**Figure 10:**
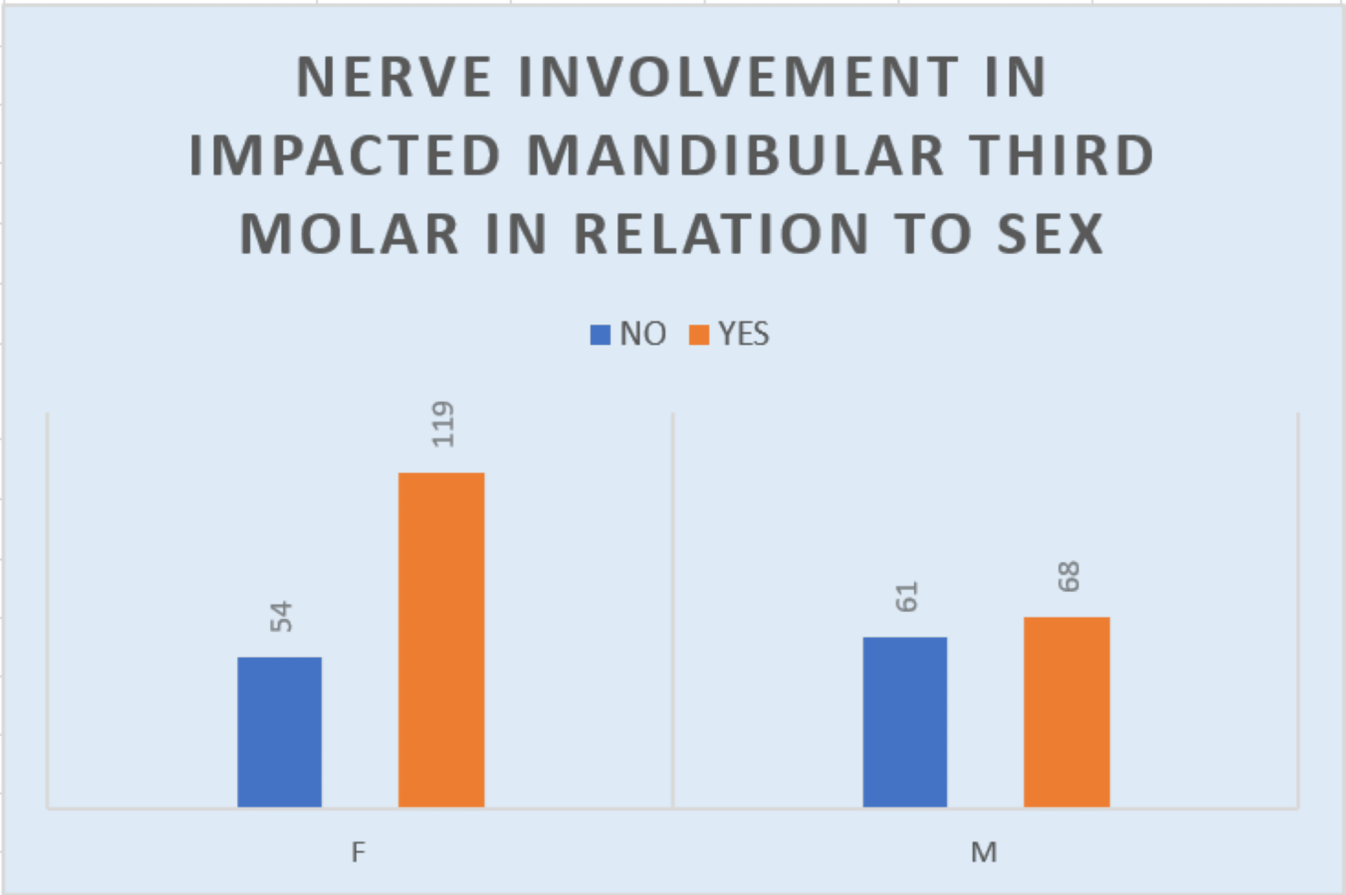
Graph describing the relation of nerve involvement to gender

When the involvement of the impacted molars with the inferior alveolar nerve (IAN) was studied according to the Rood and Shehab classification, an extensive number of 187 (61.92%) of the impacted mandibular molars showed nerve involvement (Figure 10). One hundred nineteen (39.4%) of nerve involvement was seen in women and 68 (22.51%) in men. The frequency of nerve involvement in women was 68.7% and 52.7% in men.

The most common type of nerve involvement was type 1 (n=88, 47.05%), type 5 (n=32, 17.11%), and type 4 (n=26, 13.9%). The age cohorts of 18-25, 26-35, and 56-65 all show type 1, the most common type of nerve involvement. The age group 36-45 shows types 1 and 5 to be most common with similar frequencies, and the age group 45-55 shows type 5 as the most common type of nerve involvement with type 1 in close succession (Table 8).

**Table 8:** The relation of nerve involvement to age group Statistical analysis: chi-square=62.996, df=28, and P value=0 df: degrees of freedom

Type of nerve involvement	18-25 years	26-35 years	36-45 years	46-55 years	56-65 years	Total	Percentage
1	39	31	10	4	4	88	47.05
2	6	1	0	0	0	7	3.74
3	1	5	2	1	0	9	4.81
4	9	13	2	2	0	26	13.9
5	7	8	10	5	2	32	17.11
6	6	4	2	2	2	16	8.55
7	2	5	1	1	0	9	4.81
Total	70	67	27	15	8	187	100

Sinus approximation

**Figure 11:**
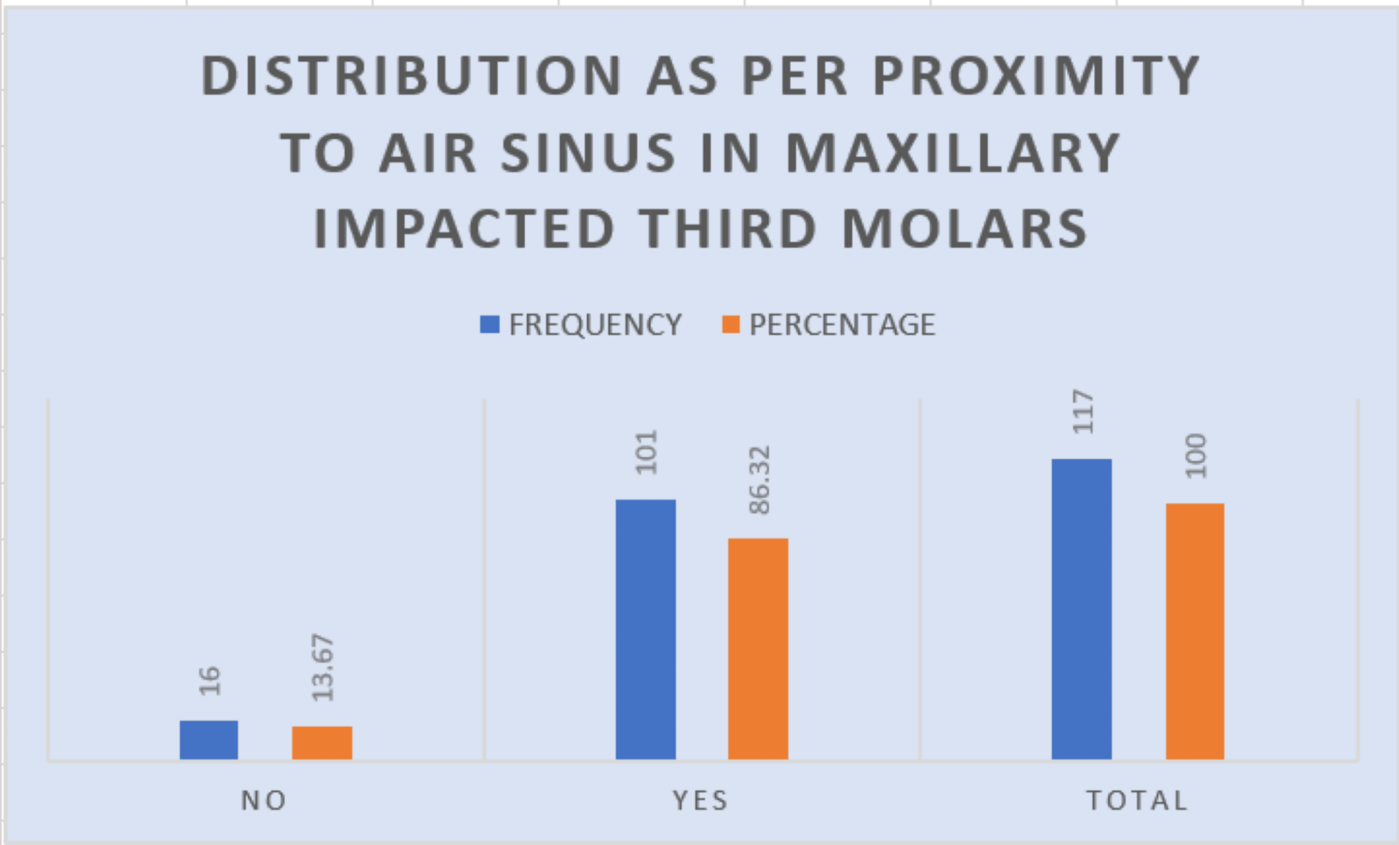
Graph describing the frequency of sinus approximation

On the investigation of the approximation of the roots of the maxillary third molars to the floor of the maxillary air sinus, 101 (86.32%) molars showed sinus approximation (Figure 11). Seventy-four (71.79%) of these were vertically impacted (Table 9).

**Table 9:** The sinus approximation and its relation to angulation Statistical analysis: chi-square=388.067, df=8, and P value=0 df: degrees of freedom

Angulation	Proximity to air sinus, no	Proximity to air sinus, yes	Total	Percentage
Distoangular	1	15	16	13.67
Horizontal	0	0	0	0
Mesioangular	5	12	17	14.52
Vertical	10	74	84	71.79
Total	16	101	117	100

Carious involvement

**Figure 12:**
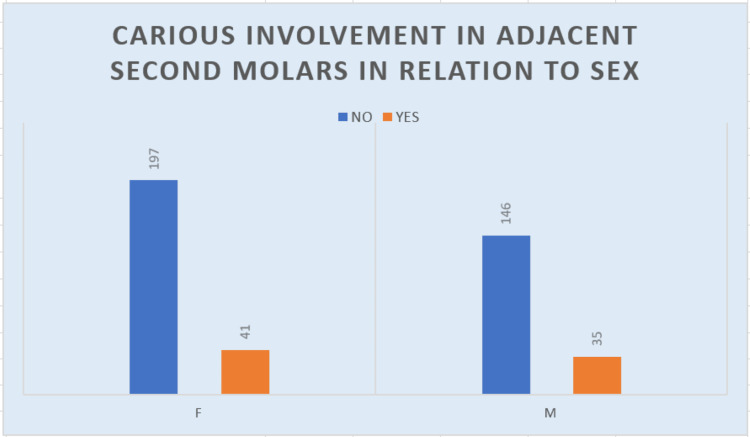
Graph describing the frequency of carious involvement

Seventy-six out of 419 (18.14%) impacted molars assessed showed the carious involvement of the adjacent second molar (Figure 12). Maximum involvement was noted in 26-35 years of age (Table 10). Of the 18.14% of impacted molars showing carious involvement, 41 (9.7%) were women, and of all molars showing the carious involvement of adjacent second molars, 41 (53.94%) were women, and 35 (46.05%) were men.

**Table 10:** Carious involvement and its relation to age group Statistical analysis: chi-square=53.056, df=8, and P value=0 df: degrees of freedom

	18-25 years	26-35 years	36-45 years	46-55 years	56-65 years	Total	Percentage
Carious involvement, no	138	103	58	29	15	343	81.86
Carious involvement, yes	16	23	19	12	6	76	18.13
Total	154	126	77	41	21	419	100

Eruption space ratio

The eruption space ratio (Figure 3), determined by dividing the mesiodistal width of the impacted third molar by the distance from the distal end of the adjacent second molar to the anterior border of the ramus, was compared with the third molar's angulation and level of impaction. This ratio in essence is comparing the space available for eruption to the space that will be required.

**Figure 13:**
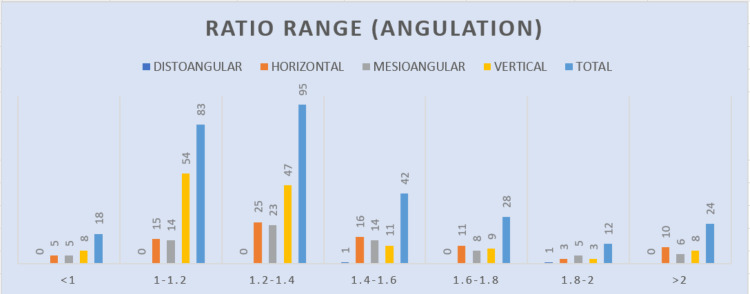
Graph describing the relation of eruption ratio to the angulation of impaction

In our observations, we found a significant relationship between the angulation of impaction and the ratio. When the ratio increases, it indicates that there is less space available for eruption. This led us to conclude that the available space affects the type and angulation of impaction that occurs (Table 11 and Figure 13).

**Table 11:** The relation of eruption ratio to the angulation of impaction Statistical analysis: chi-square=39.985, df=18, and P value=0.0021 df: degrees of freedom

Ratio range	Distoangular	Horizontal	Mesioangular	Vertical	Total	Percentage
<1	0	5	5	8	18	5.96
1-1.2	0	15	14	54	83	27.48
1.2-1.4	0	25	23	47	95	31.12
1.4-1.6	1	16	14	11	42	13.9
1.6-1.8	0	11	8	9	28	9.27
1.8-2	1	3	5	3	12	3.97
>2	0	10	6	8	24	7.945
Total	2	85	75	140	302	100

## Discussion

Third molars are the last teeth to erupt in the oral cavity. Due to this phenomenon, more than one-third of the population shows that a third molar impaction is due to insufficient available space. Our study showed the prevalence of impaction as 32.8% (419/1280). These studies are concurrent with our findings. According to Al-Madani et al.'s study published in 2024, the incidence of impaction was 33.6%, out of which 93.4% of impacted teeth are third molars [5]. On the contrary, a study done by Syed et al. showed an incidence rate of 16.31% [4].

Our study showed that vertical impactions were the most common, with more than half of impactions showing vertical angulation. All these studies also found similar findings; studies done by Al-Dajani et al. [6] and Yilmaz et al. [7] showed the presence of vertical impaction at 53.1% and 55%, respectively. However, a lot of international studies found mesioangular to be the most common type of impaction [8].

Our study found higher chances of vertical impactions in women and men as compared to other angulations and in the younger generation, i.e., 18-25 years of age. Al-Madani et al. presented the same results [5]. Ryalat et al. found that mesioangular impactions were more common in younger patients and vertical impactions as age increased [9].

Level of impaction

According to Passi et al. [2], the most frequent impaction type was class II, level B for mandibular third molars, whereas Yilmaz et al. [7] found level B (39%) to be the most common in the maxilla and level C (61%) in the mandible. For maxillary impactions, the level C pattern was most prevalent in our study. Additional research by Shaari et al. identified a higher frequency of level C impactions, which may reflect regional variations or changes in population demographics over time [10]. These trends highlight the importance of classifying impactions preoperatively to evaluate extraction complexity, anticipate complications, and plan surgical approaches accordingly. Over the past decade, most studies have consistently identified class II, level B impactions as the most common in mandibular third molars, which is concurrent with the findings of our study.

Nerve involvement

The increased risk of nerve involvement, the potential for sensory disturbances, and the proximity of the inferior alveolar nerve (IAN) to the roots of the mandibular third molars remain important concerns during extraction procedures. Sarikov and Juodzbalys found that 50% of impacted mandibular third molars were associated with significant nerve involvement, increasing the risk of postoperative sensory complications such as paresthesia [11]. Similar findings were reported by KalaiSelvan et al., with a nerve involvement rate of 67%, which is parallel with 61.92% (115/302) of molars showing nerve involvement in this study [12]. Contrastingly, a study by Kim et al. reported a higher incidence of 88%, potentially due to differences in imaging techniques or patient demographics. These results emphasize the necessity for meticulous preoperative planning and imaging using cone-beam CT (CBCT) to map the relationship between the IAN and the molar roots, allowing for appropriate surgical adjustments to minimize nerve damage [13].

Sinus approximation

The depth of impaction of the maxillary wisdom tooth is a possible predictor of the possibility of oroantral perforation if the removal of the tooth is required. This significant complication highlights the critical need for assessing the proximity of third molar roots to the maxillary sinus floor preoperatively [14]. In several studies such as ours, 86.32% (101/117) have shown a high prevalence of sinus approximation in impacted maxillary third molars, with Hadikrishna et al. showing 69.3% [15]. This reduced rate could be attributed to differences in the imaging technology. Accurate preoperative radiographic evaluation, especially using cone-beam CT (CBCT), is recommended to identify patients at risk for sinus perforation, enabling the surgeon to adopt modified surgical techniques to minimize this complication.

Carious involvement

Dental caries in second molars adjacent to impacted third molars is a common complication, particularly in younger populations. A study by Toedtling et al. reported a prevalence of 63% of radiographic distal surface caries in adjacent second molars, highlighting the importance of routine monitoring in such cases [16]. They identified some important risk factors as partially mesioangular impaction in mandibular third molars, third molars with compromised molar to the molar contact point, the loss of lamina dura, male prevalence, increasing age, and a higher caries index for the same. Our study presents a carious involvement of 18.13% (76/419), consistent with findings across studies [17]. Another study by Srivastava et al. reported a slightly higher prevalence of 37.5%, possibly due to differences in study populations or methodologies [18]. The high prevalence in younger individuals underscores the necessity for preventive measures such as fluoride treatments, improved oral hygiene education, and regular dental checkups, particularly in cases with impacted third molars. The position of the third molar, which often leads to plaque retention in hard-to-clean areas, further elevates the risk of caries in adjacent teeth.

In summary, our study aligns with previous research showing that third molar impactions are highly prevalent, with vertical impactions being the most common, particularly among younger individuals and women. The observed prevalence of impaction in our study (419/1280, 32.8%) is consistent with regional studies but contrasts with lower rates reported in other populations, suggesting possible regional or methodological differences. The limitation of this study lies in its regional scope, as third molar impaction prevalence may vary globally due to genetic, racial, and environmental factors. Conducting multicenter research with a larger, more diverse population would strengthen result validation. The predominance of vertical impactions corroborates findings from several studies, although global research indicates a higher frequency of mesioangular impactions. Class II, level B remains the most common impaction pattern, reinforcing existing trends. Overall, these findings highlight the importance of tailored preoperative planning and the need for preventive measures to manage the complexities associated with third molar extractions. The concept of the eruption space ratio, though in its initial stages, requires further research to establish its full clinical applicability. However, within the framework of this study, the concept has been introduced, paving the way for future exploration and refinement.

## Conclusions

This research provides valuable insights into the frequency, trends, and consequences of third molar impaction on adjacent molars in adults. The results highlight potential risks, such as dental caries in adjacent molars, and highlight vertical impactions as the most common type. The study also emphasizes the critical role of early radiographic screening in guiding treatment decisions and preventing complications. The introduction of the "eruption space ratio" offers a fresh perspective on the relationship between impaction and eruption space. By incorporating this parameter into existing radiographic evaluations, clinicians can better predict the difficulty of extraction and understand the surrounding bone support using a ratio-based approach. Ultimately, since third molar surgeries are part of routine minor surgeries, timely treatment is crucial for reducing the risks associated with impacted third molars, especially in younger patients and cases involving the mandibular nerve or maxillary sinus.
